# Unraveling the link: white matter damage, gray matter atrophy and memory impairment in patients with subcortical ischemic vascular disease

**DOI:** 10.3389/fnins.2024.1355207

**Published:** 2024-02-01

**Authors:** Jing Huang, Runtian Cheng, Xiaoshuang Liu, Li Chen, Tianyou Luo

**Affiliations:** ^1^Department of Radiology, The First Affiliated Hospital of Chongqing Medical University, Chongqing, China; ^2^Department of Radiology, The Affiliated Hospital of North Sichuan Medical College, Nanchong, China

**Keywords:** gray matter atrophy, magnetic resonance imaging, memory impairment, normal-appearing white matter, subcortical ischemic vascular disease, white matter hyperintensity

## Abstract

**Introduction:**

Prior MRI studies have shown that patients with subcortical ischemic vascular disease (SIVD) exhibited white matter damage, gray matter atrophy and memory impairment, but the specific characteristics and interrelationships of these abnormal changes have not been fully elucidated.

**Materials and methods:**

We collected the MRI data and memory scores from 29 SIVD patients with cognitive impairment (SIVD-CI), 29 SIVD patients with cognitive unimpaired (SIVD-CU) and 32 normal controls (NC). Subsequently, the thicknesses and volumes of the gray matter regions that are closely related to memory function were automatically assessed using FreeSurfer software. Then, the volume, fractional anisotropy (FA), mean diffusivity (MD), amplitude of low-frequency fluctuation (ALFF) and regional homogeneity (ReHo) values of white matter hyperintensity (WMH) region and normal-appearing white matter (NAWM) were obtained using SPM, DPARSF, and FSL software. Finally, the analysis of covariance, spearman correlation and mediation analysis were used to analyze data.

**Results:**

Compared with NC group, patients in SIVD-CI and SIVD-CU groups showed significantly abnormal volume, FA, MD, ALFF, and ReHo values of WMH region and NAWM, as well as significantly decreased volume and thickness values of gray matter regions, mainly including thalamus, middle temporal gyrus and hippocampal subfields such as cornu ammonis (CA) 1. These abnormal changes were significantly correlated with decreased visual, auditory and working memory scores. Compared with the SIVD-CU group, the significant reductions of the left CA2/3, right amygdala, right parasubiculum and NAWM volumes and the significant increases of the MD values in the WMH region and NAWM were found in the SIVD-CI group. And the increased MD values were significantly related to working memory scores. Moreover, the decreased CA1 and thalamus volumes mediated the correlations between the abnormal microstructure indicators in WMH region and the decreased memory scores in the SIVD-CI group.

**Conclusion:**

Patients with SIVD had structural and functional damages in both WMH and NAWM, along with specific gray matter atrophy, which were closely related to memory impairment, especially CA1 atrophy and thalamic atrophy. More importantly, the volumes of some temporomesial regions and the MD values of WMH regions and NAWM may be potentially helpful neuroimaging indicators for distinguishing between SIVD-CI and SIVD-CU patients.

## 1 Introduction

The prevalence of vascular cognitive impairment is on the rise due to the aging population, making it a significant contributor to dementia and disability in the elderly (Zhou et al., 2022). Subcortical ischemic vascular disease (SIVD) associated with aging is a primary cause of vascular cognitive impairment, attracting considerable attention in research due to its high prevalence and disability rate (Román et al., 2002). But the specific mechanisms responsible for the onset and progression of SIVD remain largely unknown. Notably, cerebrovascular disease has been identified as the second most identifiable risk factor and the only treatable factor for dementia at present (Bowler, 2005). However, the cognitive function of patients with SIVD may remain normal at an early stage, making it often difficult to identify and diagnose SIVD early (Wallin et al., 2018). Therefore, exploring the pathogenesis and early sensitive biomarkers of SIVD is essential and useful for its diagnosis and treatment.

SIVD mainly leads to executive dysfunction, but patients’ memory function can also be impaired in the early stages (Caillaud et al., 2020). Previous researches have shown that memory function was closely associated with specific gray matter regions, including the frontal cortex, temporal cortex, thalamus, amygdala, hippocampus and hippocampal subfields (Ezzyat et al., 2018; Young et al., 2018; Lee et al., 2019; Zheng et al., 2021). The abnormal alterations in the thicknesses or volumes of gray matter regions mentioned above have been identified in SIVD patients and were closely correlated with cognitive impairment (Fein et al., 2000; Cuadrado-Godia et al., 2018). However, some studies yielded inconsistent results, as researchers have not found significant volume differences in some gray matter regions between SIVD group and control group (De Guio et al., 2020; Wang et al., 2021). In addition, study indicated that cognitive impairment in patients with SIVD was also closely related to the structural damage of white matter, encompassing both white matter hyperintensity (WMH) region and normal-appearing white matter (NAWM), which were the main imaging features of SIVD (van Norden et al., 2012b). Further research also proposed that the effect of WMH volume on global cognition may be mediated by cortical atrophy (Rizvi et al., 2018). Nevertheless, the correlations of white matter structural damage, gray matter atrophy and memory impairment in patients with SIVD remains to be fully elucidated. In recent years, accumulating evidence has demonstrated the existence of functional activity in white matter, which may provide more valuable information for further understanding the pathogenesis of neuropsychiatric diseases (Ji et al., 2017; Li et al., 2020). But this aspect remains largely unexplored in patient with SIVD. Magnetic resonance imaging (MRI) technology is the main method for providing imaging evidence in patients with SIVD, enabling good evaluation of alterations in gray matter thickness and volume, while also enabling good quantification of structural and functional damages in white matter. This provides an effective way to explore the neural mechanisms of memory impairment in patients with SIVD.

This study aimed to employ MRI technology to explore the characteristic changes in white matter, gray matter and memory function in patients with SIVD, as well as the interrelationships between the three. We hypothesized that patients with SIVD may exhibit white matter damage and specific gray matter atrophy, which were closely related to memory impairment. Furthermore, it was anticipated that the relationship between white matter damage and memory impairment may be mediated through gray matter atrophy.

## 2 Materials and methods

### 2.1 Participants

A total of 90 participants, consisting of 58 patients with SIVD and 32 normal controls (NC), were enrolled between 2017 and 2021 at our hospital in this study. This study obtained ethical approval from the medical ethics committee of our hospital and informed consent from all individual participants.

Patients with SIVD were included if they met the following criteria: (1) white matter lesions: hyperintensities extending into the deep and periventricular white matter; extending caps (>10 mm as measured parallel to ventricle) or irregular halo (>10 mm and extending into deep white matter); and diffusely confluent hyperintensities (>25 mm with irregular shape) or extensive white matter lesions. (2) lacunar cases: multiple lacunas in the subcortical regions and moderate white matter lesions at least. (3) no hemorrhages, cortical and/or territorial infarcts and watershed infarcts; no signs of normal pressure hydrocephalus; and no other white matter lesions with specific causes.

Fifty-eight patients were further divided into 29 patients with cognitive impairment (SIVD-CI) and 29 patients with cognitive unimpaired (SIVD-CU). The criteria for the SIVD-CI group included: (1) subjective cognitive complaints reported by the participant or his/her caregiver; (2) objective cognitive impairments, although not meeting the Diagnostic and Statistical Manual of Mental Disorders, fourth edition (DSM-V) criteria for dementia; and (3) Clinical Dementia Rating Scale (CDR) score = 0.5 and Mini-Mental State Examination (MMSE) score = 24–26. The SIVD-CU group fulfilled the following inclusion criteria: (1) absence of subjective cognitive complaints; and (2) CDR score = 0 and MMSE score ≥ 27.

The criteria for the NC were as follows: (1) lack of neurological and psychiatric disorders; (2) lack of abnormal findings on the conventional brain MRI; and (3) lack of cognitive complaints.

The exclusion criteria for each participant included the followings: (1) metabolic conditions, such as hypothyroidism or folic acid deficiencies; (2) psychiatric and nervous system disorders, such as schizophrenia, depression, Parkinsonian syndrome, tumor or epilepsy, which can influence participant’s cognitive functions; (3) MRI scanning contraindications; and (4) inability to complete psychological scale assessment.

### 2.2 Neuropsychological assessment

Each participant underwent the following neuropsychological assessments: (1) Auditory Verbal Learning Test (AVLT) for auditory memory; (2) Rey-Osterrieth Complex Figure Test (Rey-CFT) for visual memory; (3) One-Back, digit span test (DST), and reverse digit span test (R-DST) for working memory; (4) MMSE for global cognition; (5) CDR for evaluating dementia.

### 2.3 MRI acquisition

The MRI data of all participants were gained on a GE Signa Hdxt 3.0 T scanner with an eight-channel phased-array head coil. High-resolution 3D-T1 images were acquired using the following parameters: repetition time (TR) = 8.3 ms, echo time (TE) = 3.3 ms, flip angle = 15°, slice thickness/gap = 1/0 mm, field of view (FOV) = 240 × 240 mm2, matrix = 240 × 240, resolution = 1 × 1 × 1 mm3, and scanning time = 6.45 min. The scan parameters of the T2-FLAIR weighted images were as follows: TR = 8,000 ms, TE = 126 ms, inversion time (TI) = 1,500 ms, slice thickness/gap = 5/1.5 mm, FOV = 240 × 240 mm^2^, and matrix = 256 × 192. diffusion tensor imaging (DTI) images were acquired using the following parameters: TR = 1,100 ms, TE = 77.6 ms, flip angle =15°, slice thickness/gap = 3/0 mm, FOV = 256 × 256 mm2, matrix = 128 × 128, resolution = 1 × 1 × 1 mm3, diffusion gradient encoding direction = 30, *b*-value = 1,000/0 s/mm^2^, and B0 was obtained 8 times. Resting-state functional MRI images were obtained using the following parameters: an echo-planar imaging (EPI) sequence, TR = 2,000 ms, TE = 40 ms, flip angle = 90°, slice thickness = 4 mm, FOV = 240 × 240 mm2, matrix = 64 × 64, scanning time point = 240, and scanning time = 8 min. All participants were asked to hold still, close their eyes and remain awake during the MRI scan.

### 2.4 MRI data analysis

Automatic estimations of the frontal and temporal cortical thicknesses, as well as the volumes of thalamus, amygdala, hippocampus, hippocampal subfields, and white matter, were performed on 3D-T1 images using FreeSurfer 6.0.^1^ The automatic segmentation and estimation procedures were described in previous studies (Etherton et al., 2020; Wong et al., 2021). The hippocampal subfields involved 24 regions, including the tail, subiculum, presubiculum, parasubiculum, cornu ammonis (CA)1, CA2/3, CA4, fissure, molecular layer, dentate gyrus, fimbria, and hippocampal amygdalar transition area (HATA). Previous studies have shown that the FreeSurfer automatic segmentation procedure could reliably measure the total and subfield volume of the hippocampus (Brown et al., 2020). However, it was reported that the fimbria and hippocampal fissure showed relatively low segmentation accuracies and did not belong to gray matter, so they were discarded in this study (Zhao et al., 2021). Moreover, the estimated total intracranial volume (eTIV) was obtained to adjust for the influence of brain size in subsequent statistical analysis (Etherton et al., 2020; Zeng et al., 2021). Finally, in order to guarantee the accuracy of segmentation and estimation, a thorough visual examination and manual adjustment were conducted on the segmentation and evaluation results of all participants.

The automatic segmentation and volume evaluation of WMH region were performed on T2-FLAIR images using the lesion prediction algorithm implemented in the LST toolbox version 3.0.0^2^ for Statistical Parametric Mapping 12^3^ (de Sitter et al., 2017; Iadecola et al., 2019). The detailed procedures employed in this study were reported in a previous study (de Sitter et al., 2017). Subsequently, the NAWM volume was obtained by subtracting the WMH volume from total white matter volume. Finally, the total white matter, WMH region, NAWM mask were acquired by the Data Processing Assistant for Resting-State fMRI (DPARSF) 4.3.^4^ The NAWM mask was generated by subtracting the WMH mask from the total white matter mask.

The DTI data were analyzed using FSL 6.1 software. Data processing steps included eddy current & motion correction applying the FDT tool, brain extraction applying the BET tool, and DTI measure reconstruction applying the DTI-FIT tool (Honea et al., 2022). Additionally, to evaluate the microstructural changes in white matter, the fractional anisotropy (FA) and mean diffusivity (MD) values based on WMH and NAWM mask were extracted employing Matlab code, which are widely used to evaluate the microstructural integrity of white matter fibers.

Based on previous research (Ji et al., 2017; Yang et al., 2020), the preprocessing steps of the resting-state functional MRI data with SPM12 and DPARSF 4.3 toolboxes based on MATLAB R2020b were as follows: (1) the first 10 timepoints of all participants’ functional data were removed; (2) slice-timing correction and realignment were conducted, and participants whose head motion exceeded 3.0 mm displacement or 3.0° rotation in any direction were discarded; (3) the 3D-T1 data was co-registered to the resting-state functional data and segmented into gray matter, white matter, and cerebrospinal fluid with new segments; (4) regressed 24 parameter motion correction and CSF signals; (5) generated white matter mask by a threshold (>0.9) on segmented white matter map, and then obtained white matter functional data based on white matter mask; (6) the resulting white matter functional data were normalized into a standard Montreal Neurological Institute (MNI) space by the EPI template and resampled into 3 × 3 × 3 mm^3^ voxels; (7) spatial smoothing using a 6 mm full width at half maximum Gaussian kernel, detrend and band-pass filtering (0.01–0.10 Hz) were performed in all participants. Subsequently, following a comprehensive visual examination and manual adjustment of the preprocessed data, the time series in each voxel of white matter were changed into the frequency domain, and the amplitude of low-frequency fluctuation (ALFF) and regional homogeneity (ReHo) values based on the WMH and NAWM masks were obtained (the frequency ranges from 0.01 to 0.08 Hz) using DPARSF 4.3 toolboxes. Finally, Fisher’s Z transformations were conducted on ALFF and ReHo values.

### 2.5 Statistical analysis

The statistical analyses in this study were conducted using SPSS 26.0 software and the statistical tests were corrected by Bonferroni correction for the multi-comparison. The chi-square test, Kruskal-Wallis H test, and *post-hoc* analysis were used to evaluate differences in the demographic and clinical data between groups. Furthermore, the differences in the thicknesses of frontal and temporal cortices and in the volumes of thalamus, amygdala, hippocampus, and hippocampal subfields, as well as the structural and functional indicators of white matter, were examined through analysis of covariance (ANCOVA) and *post-hoc* analysis controlling for age, gender, education and eTIV as covariates. Moreover, in order to explore the associations between the above results with significant differences and the memory scores, as well as the relationships between the white matter damage and the gray matter atrophy, the spearman correlation analyses were performed in two patient groups. Finally, to assess the correlations of the reduced memory scores, white matter damage and the specific gray matter atrophy, the mediation analyses were performed using the PROCESS SPSS macro toolbox with age, gender, education, and eTIV as covariates (Preacher and Hayes, 2004). The path coefficients and bias-corrected 95% confidence intervals (CI) were computed using bootstrap approach with 5,000 samples. The mediating effects were thought to be statistically significant if the 95% CI excluded 0. In all statistical tests, the statistical thresholds of significance were set at *p* < 0.05.

## 3 Results

### 3.1 Demographic data and memory scores

Table 1 displays the detailed comparison results of the demographic and clinical data. There was no significant difference in gender, age, education, eTIV and head motion among the three groups. All memory scores of the SIVD-CI group were significantly lower than those of the NC group (*p* < 0.05). The AVLT-IR and R-DST scores of SIVD-CU group were significantly lower than those of NC group, as well as the AVLT-IR, AVLT-DR, AVLT-RR and One-Back scores of SIVD-CI group were significantly lower than those of SIVD-CU group (*p* < 0.05).

**Table 1:** Comparison results of demographic data and memory scores between groups.

	NC (*n* = 32)	SIVD-CU (*n* = 29)	SIVD-CI (*n* = 29)	*F*/*X*^2^	*p*
Gender (M/F)	19/13	13/16	13/16	1.746	0.418^a^
Age (year)	67.50 (64.00, 71.75)	71.50 (68.50, 75.00)	70.00 (66.50, 74.50)	5.283	0.071^b^
Education (year)	9.00 (9.00, 12.00)	9.00 (9.00, 12.00)	9.00 (9.00, 12.00)	0.562	0.755^b^
eTIV (L)	1.40 ± 0.13	1.43 ± 0.13	1.42 ± 0.12	0.026	0.974^c^
Head motion	0.07 (0.04, 0.10)	0.08 (0.05, 0.10)	0.08 (0.05, 0.09)	0.433	0.805^b^
MMSE	28.50 ± 1.22	28.07 ± 0.92	24.45 ± 1.90* ^†^	74.023	<0.001^b^
AVLT-IR	8.42 ± 1.64	7.13 ± 1.76*	5.12 ± 2.21*^†^	17.386	<0.001^c^
AVLT-DR	9.31 ± 2.46	7.79 ± 2.80	5.52 ± 2.89*^†^	13.697	<0.001^c^
AVLT-RR	9.03 ± 2.98	7.45 ± 2.86	5.07 ± 2.92*^†^	13.156	<0.001^b^
Rey-CFT-IR	34.30 ± 2.17	29.45 ± 10.97	29.12 ± 9.83*	3.939	0.023^b^
Rey-CFT-DR	15.48 ± 7.35	12.47 ± 8.83	7.83 ± 7.48*	6.557	0.002^b^
DST	7.56 ± 2.17	7.93 ± 1.87	7.72 ± 1.07	2.593	0.273^b^
R-DST	4.59 ± 1.29	3.97 ± 1.38*	3.21 ± 1.78*	17.118	<0.001^b^
One-Back	57.88 ± 2.50	51.38 ± 10.88	37.38 ± 15.96*^†^	26.076	<0.001^b^

a χ^2^.

b Kruskal–Wallis H test.

c ANOVA.

^*^Significant difference compared to the NC group (*p* < 0.05).

^†^Significant difference compared to the SIVD-CU group (*p* < 0.05).

### 3.2 The thicknesses and volumes of the cortical and subcortical gray matter regions closely related to memory function

Compared with the NC group, the cortical thicknesses of the bilateral middle temporal gyrus and superior temporal gyrus were significantly decreased in the SIVD-CI group, and the cortical thicknesses of the left middle temporal gyrus and right superior temporal gyrus were significantly decreased in the SIVD-CU group (*p* < 0.05). Detailed comparison results are presented in Table 2.

**Table 2:** Comparison results of cortical thicknesses (mm) in frontal lobe and temporal lobe between groups.

	NC	SIVD-CU	SIVD-CI	*F*	*p*
**Left**					
Cortical thickness	2.46 ± 0.10	2.39 ± 0.09	2.39 ± 0.12	3.100	0.050
Frontal pole	2.66 ± 0.22	2.59 ± 0.17	2.63 ± 0.25	1.108	0.335
Caudal middle frontal	2.49 ± 0.17	2.47 ± 0.16	2.49 ± 0.16	0.251	0.778
Lateral orbito-frontal	2.67 ± 0.14	2.60 ± 0.12	2.65 ± 0.20	1.634	0.201
Medal orbito-frontal	2.46 ± 0.11	2.38 ± 0.12	2.33 ± 0.60	1.891	0.157
Rostral middle frontal	2.33 ± 0.13	2.28 ± 0.11	2.28 ± 0.15	1.320	0.273
Superior frontal	2.66 ± 0.15	2.62 ± 0.13	2.61 ± 0.16	0.814	0.447
Inferior temporal	2.89 ± 0.16	2.81 ± 0.15	2.81 ± 0.18	1.684	0.192
Middle temporal	2.86 ± 0.10	2.77 ± 0.14*	2.71 ± 0.15*	10.046	<0.001
Superior temporal	2.64 ± 0.14	2.55 ± 0.13	2.53 ± 0.18*	3.781	0.027
Temporal pole	3.63 ± 0.26	3.49 ± 0.22	3.47 ± 0.33	2.266	0.110
Transverse temporal	2.32 ± 0.21	2.22 ± 0.20	2.24 ± 0.18	1.689	0.191
**Right**					
Cortical thickness	2.46 ± 0.10	2.39 ± 0.09	2.39 ± 0.12	3.054	0.052
Frontal pole	2.62 ± 0.26	2.61 ± 0.18	2.56 ± 0.25	0.518	0.598
Caudal middle frontal	2.50 ± 0.14	2.48 ± 0.18	2.51 ± 0.20	0.224	0.800
Lateral orbito-frontal	2.67 ± 0.16	2.62 ± 0.15	2.59 ± 0.23	1.506	0.228
Medal orbito-frontal	2.50 ± 0.15	2.50 ± 0.15	2.48 ± 0.19	0.405	0.668
Rostral middle frontal	2.34 ± 0.16	2.28 ± 0.11	2.29 ± 0.13	1.572	0.214
Superior frontal	2.65 ± 0.13	2.61 ± 0.14	2.62 ± 0.18	0.229	0.796
Inferior temporal	2.85 ± 0.16	2.78 ± 0.14	2.79 ± 0.19	1.580	0.212
Middle temporal	2.83 ± 0.12	2.76 ± 0.14	2.73 ± 0.16*	3.647	0.030
Superior temporal	2.68 ± 0.13	2.54 ± 0.15*	2.56 ± 0.16*	6.717	0.002
Temporal pole	3.67 ± 0.27	3.59 ± 0.27	3.58 ± 0.29	1.019	0.366
Transverse temporal	2.34 ± 0.22	2.27 ± 0.21	2.28 ± 0.20	1.165	0.317

*Significant difference compared to the NC group (*p* < 0.05), †: significant difference compared to the SIVD-CU group (*p* < 0.05).

**Figure 1:**
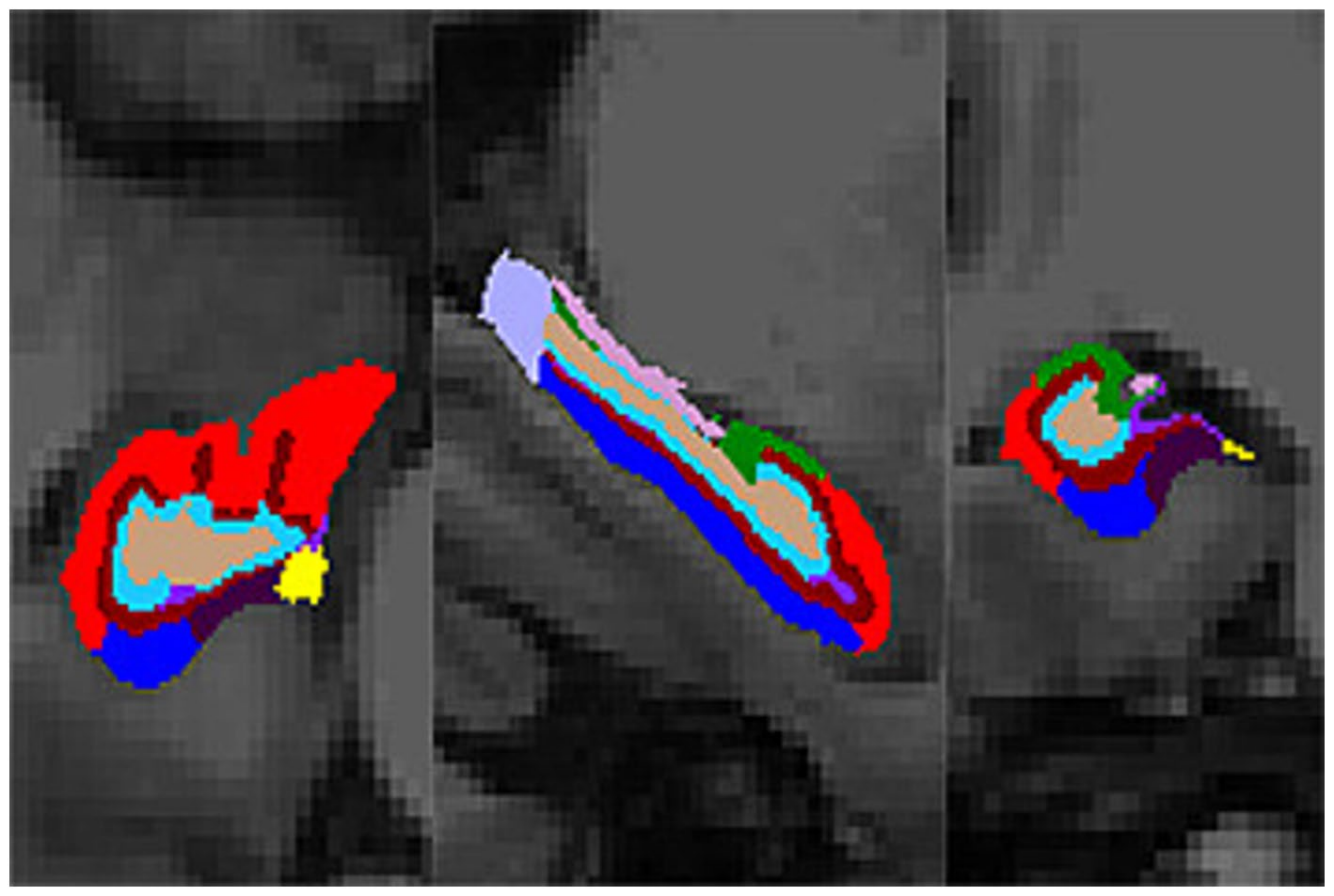
Hippocampal subfield segmentation result (transverse, sagittal, and coronal views). Color code: red, CA1; dark green, CA2/3; light green, HATA; light brown, CA4; dark brown, molecular layer; rose red, fimbria; dark blue, subiculum; light blue, dentate gyrus; light purple, hippocampal tail; moderate purple, hippocampal fissure; dark purple, presubiculum; yellow, parasubiculum.

Compared with the NC group, the volumes of the bilateral thalamus, amygdala, hippocampus, CA1, subiculum, presubiculum, molecular layer and dentate gyrus were significantly decreased in the SIVD-CI group, and the volumes of the bilateral thalamus, left amygdala, left hippocampus, left CA1, left CA2/3, left CA4, bilateral molecular layer and left dentate gyrus were significantly decreased in the SIVD-CU group (*p* < 0.05). Compared with the SIVD-CU group, the volumes of the left CA2/3, right amygdala and right parasubiculum were significantly decreased in the SIVD-CI group (*p* < 0.05). Detailed comparison results are presented in Table 3. Figure 1 displays the left hippocampal subfield segmentation result for one NC subject.

**Table 3:** Comparison results of thalamus, amygdala, hippocampus, and hippocampal subfield volumes (mm3) between groups.

	NC	SIVD-CU	SIVD-CI	*F* value	*p* value
**Left**					
Thalamus	6142.69 ± 652.49	5764.82 ± 557.67*	5696.52 ± 748.94*	6.399	0.003
Amygdala	1467.86 ± 181.46	1321.20 ± 185.97*	1288.26 ± 199.09*	9.874	<0.001
Hippocampus	3143.10 ± 265.51	2926.12 ± 327.24*	2995.88 ± 273.24*	6.218	0.003
CA1	579.02 ± 61.03	548.77 ± 65.98*	548.76 ± 45.45*	6.108	0.003
CA2-3	184.69 ± 18.28	163.56 ± 25.95*	178.68 ± 23.95^†^	7.848	0.001
CA4	232.23 ± 21.25	211.66 ± 25.10*	221.29 ± 21.89	7.299	0.001
Tail	480.35 ± 71.87	444.28 ± 75.04	456.25 ± 58.81	2.394	0.098
Subiculum	403.27 ± 42.10	382.90 ± 45.54	373.64 ± 46.65*	3.915	0.024
Presubiculum	287.39 ± 36.57	274.11 ± 34.62	270.13 ± 40.13*	2.731	0.071
Parasubiculum	62.13 ± 12.80	62.75 ± 16.30	65.53 ± 18.49	0.296	0.745
Molecular layer	514.22 ± 44.09	476.06 ± 53.01*	482.92 ± 46.43*	7.319	0.001
Dentate gyrus	266.95 ± 26.30	239.97 ± 27.79*	249.19 ± 26.15*	9.375	<0.001
HATA	67.87 ± 89.03	51.81 ± 8.06	53.51 ± 13.01	0.657	0.521
**Right**					
Thalamus	5855.09 ± 625.90	5526.69 ± 520.59*	5422.05 ± 722.03*	5.975	0.004
Amygdala	1634.24 ± 218.00	1604.52 ± 200.10	1471.66 ± 213.51*^†^	6.788	0.002
Hippocampus	3240.75 ± 288.69	3097.59 ± 314.29	3014.45 ± 307.70*	7.385	0.001
CA1	606.02 ± 58.19	578.96 ± 55.06	574.74 ± 59.60*	5.713	0.005
CA2-3	199.04 ± 23.49	190.70 ± 24.66	188.82 ± 31.33	1.796	0.172
CA4	241.98 ± 22.38	233.91 ± 23.92	231.44 ± 31.36	2.198	0.117
Tail	498.13 ± 76.30	467.41 ± 84.96	466.11 ± 62.27	2.874	0.062
Subiculum	412.80 ± 40.68	390.89 ± 38.46	376.38 ± 43.82*	7.679	0.001
Presubiculum	277.64 ± 33.19	259.33 ± 37.15	243.33 ± 38.68*	9.129	<0.001
Parasubiculum	60.93 ± 11.04	63.13 ± 15.76	53.28 ± 16.03^†^	4.004	0.022
Molecular layer	530.92 ± 46.81	503.35 ± 49.58*	490.00 ± 51.41*	8.462	<0.001
Dentate gyrus	279.01 ± 28.76	264.63 ± 25.70	261.13 ± 35.55*	4.188	0.019
HATA	53.85 ± 7.88	54.91 ± 8.43	51.28 ± 10.41	1.944	0.150

*Significant difference compared to the NC group (*p* < 0.05), †: significant difference compared to the SIVD-CU group (*p* < 0.05).

### 3.3 The volume, FA and MD values of WMH region and NAWM

**Figure 2:**
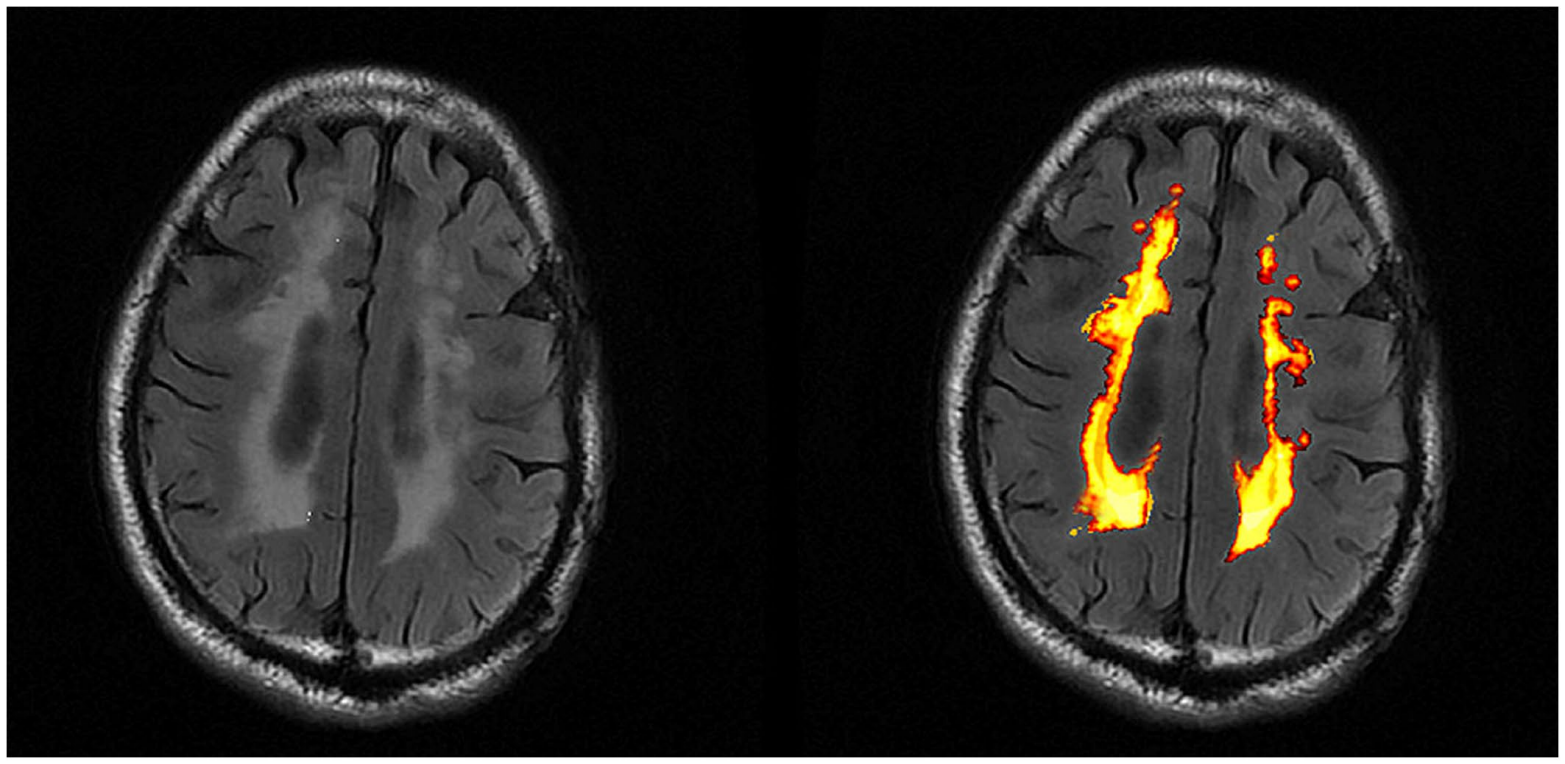
WMH segmentation result (transverse views).

Compared with the NC group, the significant increase of WMH volume and the significant reductions of FA values in the WMH region and NAWM were found in SIVD-CI and SIVD-CU group, and the significant increases of MD values and the significant reductions of ALFF and ReHo values in the WMH region and NAWM were observed in the SIVD-CI group (*p* < 0.05). Compared with the SIVD-CU group, the significant reductions of NAWM volume and the significant increases of MD values in the WMH region and NAWM were found in the SIVD-CI group (*p* < 0.05). Detailed comparison results are presented in Table 4. Figure 2 displays the WMH segmentation results of a patient with SIVD.

**Table 4:** Comparison results of volume (ml), FA, MD, ALFF, and ReHo values in WMH region and NAWM between groups.

	NC	SIVD-CU	SIVD-CI	*F* value	*p* value
WMH volume	3.06 ± 2.63	29.31 ± 12.02*	33.84 ± 14.73*	73.249	<0.001
WMH-FA	0.2913 ± 0.0382	0.2339 ± 0.0243*	0.2269 ± 0.0271*	44.656	<0.001
WMH-MD	0.0011 ± 0.0001	0.0011 ± 0.0001	0.0012 ± 0.0001*^†^	7.870	0.001
WMH-ALFF	0.9743 ± 0.5920	0.7025 ± 0.1465*	0.6823 ± 0.1309*	4.759	0.012
WMH-ReHo	0.9889 ± 0.0547	0.9705 ± 0.0251*	0.9626 ± 0.0286	3.384	0.040
NAWM volume	410.19 ± 45.70	367.90 ± 44.35*	344.66 ± 42.84*^†^	43.721	<0.001
NAWM-FA	0.2238 ± 0.01984	0.1956 ± 0.0164*	0.1914 ± 0.0150*	38.371	<0.001
NAWM-MD	0.0010 ± 0.0001	0.0010 ± 0.0001	0.0011 ± 0.0001*^†^	15.254	<0.001
NAWM-ALFF	1.1571 ± 0.8202	0.9647 ± 0.2165	0.9334 ± 0.1880	1.268	0.288
NAWM-ReHo	0.9437 ± 0.0305	0.9391 ± 0.0294	0.9519 ± 0.0279	1.299	0.280

^*^Significant difference compared to the NC group (*p* < 0.05).

^†^Significant difference compared to the SIVD-CU group (*p* < 0.05).

### 3.4 Correlation analysis

For the SIVD-CI group, the right thalamus volume was positively correlated with AVLT-IR scores (*r* = 0.432, *p* = 0.045); the cortical thickness in right middle temporal gyrus (*r* = 0.417, *p* = 0.031) and left middle temporal gyrus (*r* = 0.413, *p* = 0.040) were positively correlated with AVLT-RR scores; the right CA1 volume was positively correlated with Rey-CFT-IR scores (*r* = 0.447, *p* = 0.028); the right presubiculum volume was negatively associated with AVLT-IR (*r* = −0.440, *p* = 0.028) and AVLT-RR scores (*r* = −0.487, *p* = 0.014); the left molecular layer (*r* = 0.407, *p* = 0.048) and dentate gyrus (*r* = 0.496, *p* = 0.014) volumes were positively correlated with Rey-CFT-IR scores; the left molecular layer (*r* = 0.417, *p* = 0.024) and right presubiculum (*r* = 0.422, *p* = 0.022) volumes were positively correlated with R-DST scores; the right thalamus volume was negatively correlated with the MD values in WMH region (*r* = −0.479, *p* = 0.010) and in NAWM (*r* = −0.432, *p* = 0.025); the cortical thicknesses in right middle temporal gyrus (*r* = −0.437, *p* = 0.023) and left middle temporal gyrus (*r* = −0.465, *p* = 0.015) were negatively correlated with the MD value in WMH region; the right CA1 volume was negatively correlated with the FA value in WMH region (*r* = −0.540, *p* = 0.002); the right presubiculum volume was negatively correlated with the MD value in WMH region (*r* = −0.453, *p* = 0.014); the MD values in WMH region (*r* = −0.449, *p* = 0.015) and NAWM (*r* = −0.480, *p* = 0.008) were negatively correlated with the R-DST scores; the ReHo value in WMH region was positively correlated with the R-DST scores (*r* = 0.559, *p* = 0.007).

**Figure 3:**
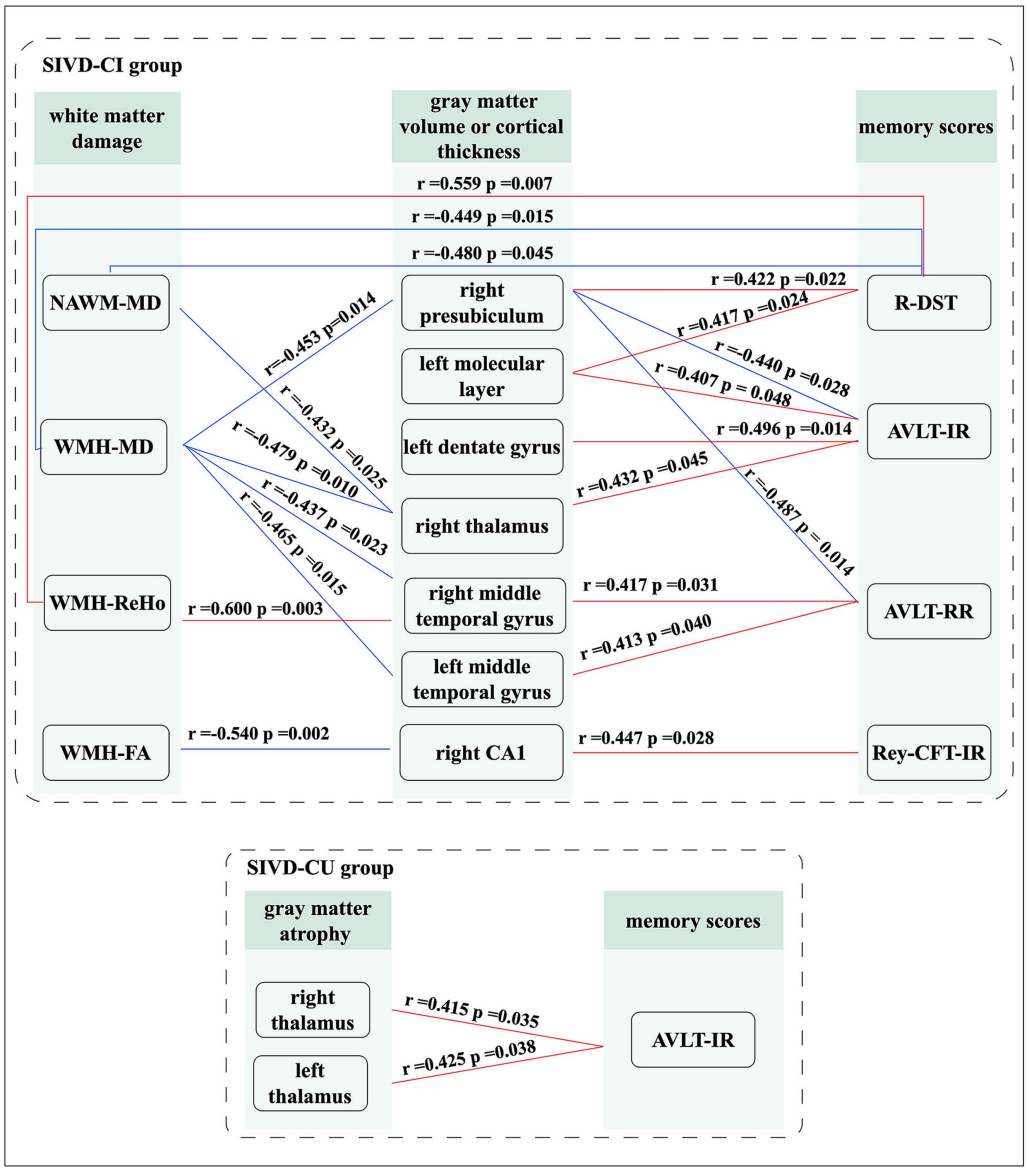
Significant correlation analysis results. Red lines: represents positive correlation; Blue lines: represents negative correlation.

For the SIVD-CU group, the right thalamus (*r* = 0.415, *p* = 0.035) and left thalamus (*r* = 0.425, *p* = 0.038) volumes were positively correlated with Rey-CFT-IR scores. All significant correlation analysis results are shown in Figure 3.

### 3.5 Mediation analysis

**Figure 4:**
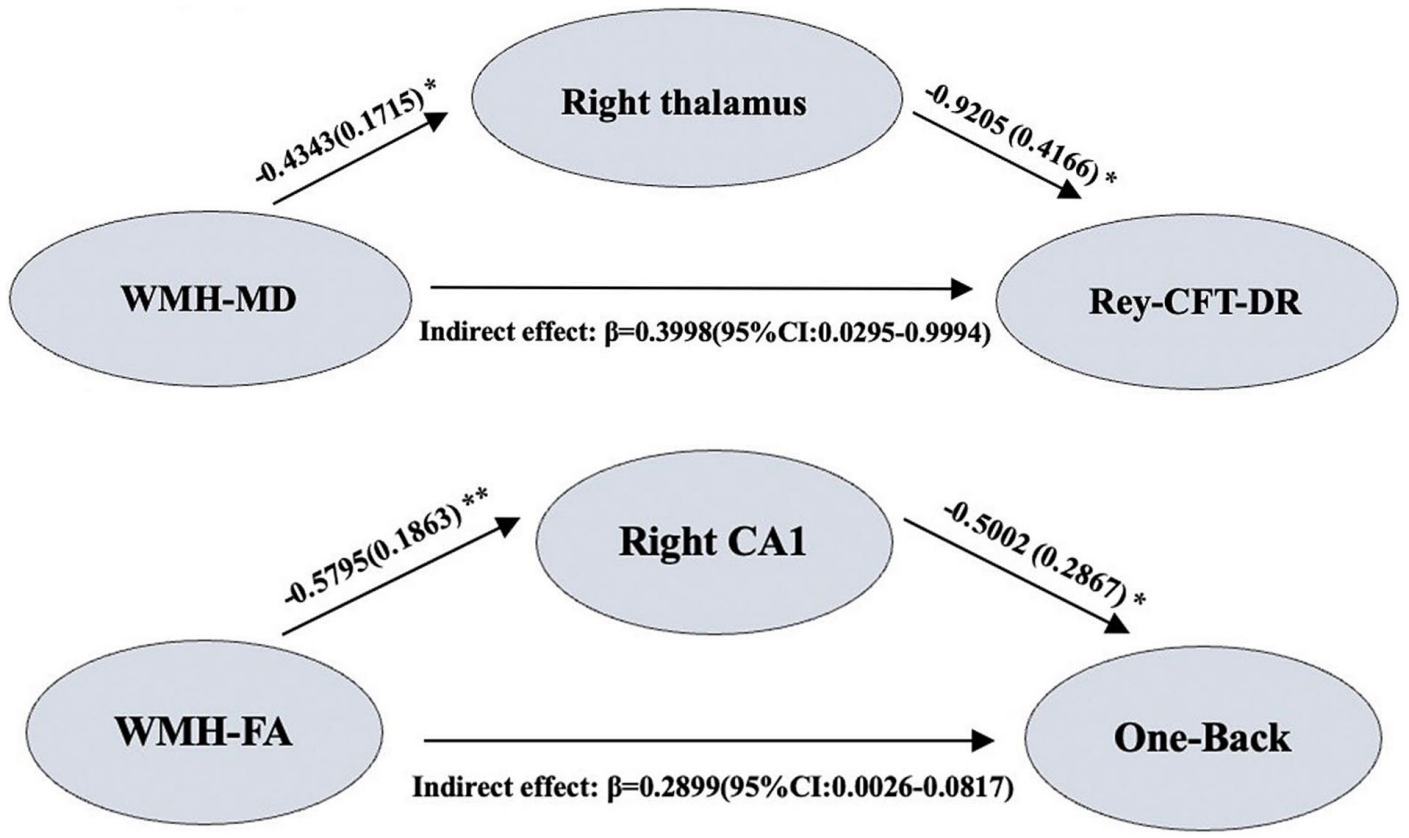
Mediation analysis results. *p < 0.05, **p < 0.01.

Our study explored whether the gray matter atrophy mediates the relationship between white matter damage and memory decline. As illustrated in Figure 4, we found that the association between the decreased FA value in WMH region and the decreased One-Back scores was mediated by the decreased volume of right CA1 [β = 0.2899, 95% bootstrap CI (0.0026, 0.0817)], and the association between the increased MD value in WMH region and the decreased Rey-CFT-DR scores was mediated by the decreased volume of right thalamus [β = 0.3998, 95% bootstrap CI (0.0295, 0.9994)] in the SIVD-CI group. However, there was no mediator association in the SIVD-CU group and the NC group.

## 4 Discussion

Our study investigated the changes in memory function, white matter and gray matter in patients with SIVD, as well as their relationship. The primary findings were summarized as follows: (1) The auditory memory, visual memory, and working memory scores in the SIVD-CI group were significantly lower than those in the NC group. (2) Significant gray matter atrophy was observed in patients with SIVD, mainly including the thalamus, amygdala, superior temporal gyrus, middle temporal gyrus, hippocampus and hippocampal subfields such as CA1, subiculum, presubiculum, motor layer and dentate gyrus. The atrophic brain regions in the SIVD-CI group were more extensive than those in the SIVD-CU group. Furthermore, compared with the SIVD-CU group, the significant reductions of the left CA2/3, right amygdala and right parasubiculum volumes were found in the SIVD-CI group, but these findings were not correlated with memory scores. (3) Compared with the NC group, the SIVD-CI and SIVD-CU groups exhibited significant volume, FA, ALFF and ReHo value decreases, as well as MD value increase in WMH region and NAWM. Compared with the SIVD-CU group, the significant reductions of the NAWM volumes and the significant increases of the MD values in the WMH region and NAWM were found in the SIVD-CI group. (4) In the patient group, the decreased volumes of the right thalamus, right CA1, right presubiculum, left molecular layer and left dentate gyrus, as well as the decreased cortical thickness of the left middle temporal gyrus, were significantly correlated with the decreased memory scores; the decreased FA and ReHo values and increased MD values in WMH region and NAWM were significantly correlated with the decreased memory scores and some decreased gray matter volumes. (5) Mediation analysis revealed that the diminished volumes of the right CA1 and right thalamus mediated the correlations between the abnormal FA and MD values in WMH region and the decreased visual memory and working memory scores.

### 4.1 Memory impairment

SIVD mainly leads to executive dysfunction, but more and more studies have shown that SIVD may affect a wide range of cognitive domains, including memory function (Reed et al., 2007; Caillaud et al., 2020). Our study found significant reductions in auditory memory, visual memory and working memory scores in patients with SIVD compared with the NC. Our findings were largely consistent with the findings of previous studies (Palesi et al., 2018; Feng et al., 2021). In addition, He et al. (2022) discovered that memory function decreased with the decline of global cognitive function in patients with SIVD. Similarly, the present study revealed that the auditory and working memory scores of SIVD-CU group were significantly lower than those of NC group, and were significantly higher than those of SIVD-CI group. This indicated that patients with SIVD had auditory memory and working memory deficits in the early stage, and may progressively worsen as global cognitive function deteriorates.

### 4.2 White matter damage and its relationship with memory impairment

As expected, significant reductions in FA, ALFF, and ReHo values in the WMH region and NAWM, as well as a significant increase in MD values and volumes in the WMH region, were observed in the patients of SIVD-CI group and SIVD-CU group in this study. This indicated that SIVD patients exhibited extensive macrostructural, microstructural and functional damages in white matter. Prior studies aligned with our results, which further revealed that the abnormal changes of WMH region and NAWM were related to memory impairment in SIVD patients (Xu et al., 2010; Rizvi et al., 2020). Zeng et al. (2020) found that the severity of WMH was significantly negatively correlated with working memory scores. The brain regions communicate information through structurally and functionally intact white matter fibers, while the normal working memory function relies on normal information communications in the medial temporal and prefrontal systems (Constantinidis and Procyk, 2004). More importantly, compared with the SIVD-CU group, significant NAWM atrophy and significant microstructural damage in the WMH region and NAWM were observed in the SIVD-CI group in our study, and these white matter microstructure injuries were significantly correlated with the decrease of working memory scores. Hence, the microstructural injuries of WMH and NAWM may be key imaging markers for cognitive impairment in SIVD patients, which may help distinguish between SIVD-CI and SIVD-CU patients in the future. Unexpectedly, we did not find any significant association between the memory scores and the WMH and NAWM volume in SIVD patients. This finding was consistent with previous research result, suggesting that DTI indicators may serve as more sensitive indicators of memory impairment in SIVD patients, surpassing conventional MRI indicators such as volume (Xu et al., 2010). Similarly, van Norden et al. (2012a) also shown that DTI may be a promising tool for exploring the underlying mechanisms of memory decline in patients with SIVD.

### 4.3 Gray matter atrophy and its relationship with white matter damage and memory impairment

Brain atrophy is a common occurrence in elderly individuals and tends to worsen with age. The cerebral ischemia and hypoxia can also contribute to the development and progression of brain atrophy. Our study results indicated that patients in SIVD-CI group showed extensive atrophy in gray matter regions that are closely related to memory function, mainly involving the thalamus, amygdala, superior temporal gyrus, middle temporal gyrus, hippocampus and specific hippocampal subfields such as CA1, subiculum, presubiculum, motor layer and dentate gyrus. The atrophic gray matter regions observed in SIVD-CU group were similar to those in the SIVD-CI group, but relatively fewer, which is the prodromal stage of SIVD. This suggested that patients with SIVD have already exhibited specific atrophy in gray matter regions closely related to memory function in the early stages. Previous studies have yielded consistent results, finding that the volumes of frontal lobe, temporal lobe, hippocampus, thalamus and amygdala in SIVD patients were significantly reduced compared to normal individuals, indicating widespread cortical and subcortical gray matter atrophy (Seo et al., 2010; Tan et al., 2023). Additionally, a multicenter study using cross-sectional and longitudinal data further found that cortical and subcortical atrophy may be independent influencing factors for cognitive decline in SIVD patients, and may exacerbate the influence of white matter lesions on cognitive dysfunction (Jokinen et al., 2012). To some extent, the present study supports this finding, demonstrating that the atrophies of thalamus, CA1, presubiculum and bilateral middle temporal gyrus were significantly correlated with the structural and functional damages of WMH region and NAWM in SIVD-CI group. The vulnerability of the brain’s white matter to ischemia renders it prone to neuronal necrosis, axonal loss and demyelination, which impedes communication between the cortical and subcortical gray matter regions, ultimately leading to their degeneration and atrophy (Molko et al., 2001). Consequently, it can be inferred that white matter structural and functional damages may be a partial mechanism of brain atrophy in patients with SIVD, but more research is needed to validate this inference.

The thalamus is a pivotal subcortical gray matter in the brain, as it is a central relay station connecting other gray matter regions (Mai and Majtanik, 2018). Its primary function involves the processing and regulation of sensory information, especially auditory information (Mai and Majtanik, 2018). In the current study, we observed significant associations between the thalamic atrophy with the decreased auditory and visual memory scores in SIVD-CI group and SIVD-CU group. A recent study, which was conducted on individuals with mild cognitive impairment, showed a close correlation between the functional connectivity disruption in thalamus and the impaired memory function (Kim et al., 2021). Therefore, the injury of thalamus in SIVD patients with or without cognitive impairment may be an important cause of their auditory and visual memory impairment. In addition, we found that the right thalamic atrophy mediated the relationship between the increased MD value of WMH region and the visual memory impairment in SIVD-CI group. Kim et al. (2021) supported our findings from different perspectives, finding that the impact of WMH region on memory function may be mediated by the interrupted structural and functional connectivity of white matter. This indicates that the microstructural damage in WMH region may also have an indirect effect on memory function through subcortical gray matter atrophy such as the thalamus, which may reflect the neurodegeneration of gray matter caused by axonal damage related to SIVD, exacerbating cognitive impairment in turn (Rizvi et al., 2018). However, a recent study about small vessel disease patients without thalamic lacunes displayed that thalamocortical MD values mediated the relationship between thalamic volume and memory function, while thalamic volume exhibited no mediating effects in the relations between the thalamocortical MD values and memory function (Li et al., 2023). The reasons for the inconsistent results may be due to different study cohorts or different data analysis methods. But this may support our findings from another perspective, that is, thalamic atrophy may also indirectly impair cognitive function by affecting its structural connections with the cortex. In summary, the thalamic atrophy may play a crucial role in auditory and visual memory impairment in patients with SIVD.

The temporal lobe plays a crucial role in the normal memory function and the processing of auditory information (Tan et al., 2023). Our study observed significant associations between the atrophy of left middle temporal gyrus and the decreased auditory memory scores in SIVD-CI group. Sun et al. (2022) also found a negative correlation between the temporal lobe atrophy and the decreased situational memory scores in cerebral small vessel disease patients with cognitive impairment. Moreover, a recent longitudinal study on 477 participants reported that the temporal lobe atrophy was closely related to longitudinal memory decline in SIVD patients (Jokinen et al., 2012). These findings suggested that temporal lobe atrophy may serve as a neural mechanism underlying memory dysfunction in patients with SIVD. Notably, the hippocampus is an important component of the temporal lobe, consisting of several subfields such as CA, subiculum, presubiculum, parasubiculum, dentate gyrus, and molecular layer (Brown et al., 2020). An autopsy study found reduced volume and neuronal loss in the hippocampus in SIVD patients with dementia (Kril et al., 2002). Li et al. (2016) further observed that patients with mild subcortical vascular cognitive impairment exhibited significant atrophy in the subiculum, presubiculum and dentate gyrus. In addition, research has shown that the hippocampal subfield volumes may be more specific and sensitive neuroimaging markers for dementia than the global hippocampal volume or medial temporal lobe volume (Dubois et al., 2007). We provide further evidences for this finding, demonstrating that the volume atrophy of the right CA1, right presubiculum, left motor layer, and left dentate gyrus were associated with decreased visual and working memory scores in SIVD-CI group. Wong et al. (2021) also found that the atrophy of CA1, CA4, molecular layer and dentate gyrus were significantly associated with memory decline. As important nodes in the hippocampal neural circuit, the presubiculum, molecular layer and dentate gyrus receive information input from multiple cortical regions, which were related to normal memory performance (Wong et al., 2021). CA1 is involved in the formation of human brain memory, but is extremely sensitive to ischemia, and its damage has been proven to be related to poor memory function (Mueller et al., 2011). An animal study found that CA1 played an important role in working memory and spatial memory information processing (Ahnaou et al., 2017). Our results also revealed that the right CA1 volume reduction mediated the impact of WMH microstructure damage on working memory decline in SIVD-CI group. Similarly, Rizvi et al. (2018) observed that hippocampal volume acted as a mediator in the associations between the WMH volume and the global cognitive and memory function. However, van Leijsen et al. (2019) did not observe a mediating effect of hippocampal atrophy, this may be explained by different data analysis methods. To summarize, the memory impairment in SIVD patients may be associated with specific hippocampal subfield atrophy. It is worth mentioning that compared with the SIVD-CU group, the significant atrophy of the left CA2/3, right amygdala and right parasubiculum were found in the SIVD-CI group, but these changes were not significantly correlated with memory scores. The CA2/3, amygdala and parasubiculum are important components of the cognitive circuits and have significance in maintaining normal cognition (Hitti and Siegelbaum, 2014; Yang and Wang, 2017). Therefore, to some extent, the atrophy of temporomesial regions may also help distinguish between SIVD-CI and SIVD-CU patients and large-scale or longitudinal studies are needed to verify it.

### 4.4 Study limitations

Several limitations of the current study must be mentioned. Firstly, this cross-sectional study had a relatively small sample size and did not include patients with dementia, so longitudinal studies and additional studies in a larger sample are needed to confirm the current results. Secondly, this study only considered the structural and functional changes of WMH region and NAWM, but the spatial distribution of WMH region and NAWM, as well as other traditional MRI indicators of SIVD such as lacunae, are also crucial for cognitive outcomes. Thirdly, our study could not completely exclude patients with mixed Alzheimer’s disease and vascular pathology. In the future, it is necessary to exclude SIVD patients with Alzheimer’s disease as much as possible.

## 5 Conclusion

This study found that patients with SIVD exhibited specific gray matter atrophy as well as structural and functional abnormalities in WMH region and NAWM, which were closely related to memory impairment, especially CA1 and thalamic atrophy. Our findings may help to the advancement of our comprehension regarding the neural mechanisms underlying memory impairment, and also could providing specific neuroimaging markers for the early diagnosis of memory impairment in patients with SIVD. More importantly, the volumes of some temporomesial regions and the MD values of WMH regions and NAWM may be potentially helpful neuroimaging indicators for distinguishing between SIVD-CI and SIVD-CU patients.

## Data availability statement

The raw data supporting the conclusions of this article will be made available by the authors, without undue reservation.

## Ethics statement

The studies involving humans were approved by the medical ethics committee of the First Affiliated Hospital of Chongqing Medical University. The studies were conducted in accordance with the local legislation and institutional requirements. The participants provided their written informed consent to participate in this study. Written informed consent was obtained from the individual(s) for the publication of any potentially identifiable images or data included in this article.

## Author contributions

JH: Investigation, Methodology, Software, Visualization, Writing – original draft, Writing – review & editing. RC: Formal analysis, Methodology, Software, Validation, Writing – review & editing. XL: Formal analysis, Methodology, Software, Validation, Writing – review & editing. LC: Formal analysis, Methodology, Software, Validation, Writing – review & editing. TL: Conceptualization, Data curation, Formal analysis, Funding acquisition, Methodology, Project administration, Resources, Supervision, Validation, Writing – review & editing.

## Footnotes

^1^ http://surfer.nmr.mgh.harvard.edu

^2^ www.statistical-modelling.de/lst.html

^3^ http://www.fil.ion.ucl.ac.uk/spm

^4^ http://rfmri.org/DPARSF
